# User Engagement and Clinical Impact of the Manage My Pain App in Patients With Chronic Pain: A Real-World, Multi-site Trial

**DOI:** 10.2196/26528

**Published:** 2021-03-04

**Authors:** Anuj Bhatia, Jamal Kara, Tahir Janmohamed, Atul Prabhu, Gerald Lebovic, Joel Katz, Hance Clarke

**Affiliations:** 1 Department of Anesthesia and Pain Medicine University Health Network University of Toronto Toronto, ON Canada; 2 Institute of Health Policy Management and Evaluation University of Toronto Toronto, ON Canada; 3 Department of Anesthesia and Pain Management Toronto Western Hospital University Health Network Toronto, ON Canada; 4 Institute of Psychiatry, Psychology, and Neuroscience King's College London London United Kingdom; 5 ManagingLife Inc Toronto, ON Canada; 6 Applied Health Research Centre Unity Health Toronto Toronto, ON Canada; 7 Department of Psychology York University Toronto, ON Canada; 8 Transitional Pain Service Toronto General Hospital University Health Network Toronto, ON Canada

**Keywords:** pain, psychology, patient-oriented research, quality of life, digital health, chronic pain, pain app, virtual care, mHealth, pain management, chronic disease management, remote monitoring, app, engagement, impact, outcome

## Abstract

**Background:**

Chronic pain imposes a large burden on individuals and society. A patient-centric digital chronic pain management app called Manage My Pain (MMP) can be used to enhance communication between providers and patients and promote self-management.

**Objective:**

The purpose of this study was to evaluate the real-world engagement of patients in urban and rural settings in Ontario, Canada with the MMP app alongside their standard of care and assess the impact of its usage on clinical outcomes of pain and related mental health.

**Methods:**

A total of 246 participants with chronic pain at a rural and 2 urban pain clinics were recruited into this prospective, open-label, exploratory study that compared the use of MMP, a digital health app for pain that incorporates validated questionnaires and provides patients with summarized reports of their progress in combination with standard care (app group), against data entered on paper-based questionnaires (nonapp group). Participants completed validated questionnaires on anxiety, depression, pain catastrophizing, satisfaction, and daily opioid consumption up to 4.5 months after the initial visit (short-term follow-up) and between 4.5 and 7 months after the initial visit (long-term follow-up). Engagement and clinical outcomes were compared between participants in the two groups.

**Results:**

A total of 73.6% (181/246) of the participants agreed to use the app, with 63.4% (111/175) of them using it for at least one month. Individuals who used the app rated lower anxiety (reduction in Generalized Anxiety Disorder 7-item questionnaire score by 2.10 points, 95% CI –3.96 to –0.24) at short-term follow-up and had a greater reduction in pain catastrophizing (reduction in Pain Catastrophizing Scale score by 5.23 points, 95% CI –9.55 to –0.91) at long-term follow-up relative to patients with pain who did not engage with the MMP app.

**Conclusions:**

The use of MMP by patients with chronic pain is associated with engagement and improvements in self-reported anxiety and pain catastrophizing. Further research is required to understand factors that impact continued engagement and clinical outcomes in patients with chronic pain.

**Trial Registration:**

ClinicalTrials.gov NCT04762329; https://clinicaltrials.gov/ct2/show/NCT04762329

## Introduction

Several large population-based surveys show that over 1 in 5 people live with chronic pain [1-5]. Pain is associated with poor quality of life [6] and is one of the top 3 reasons to seek medical attention in Canada [7,8]. The combined direct and indirect annual costs of chronic pain in North America are estimated to be more than US $650 billion [6,9-11]. Despite these staggering numbers, there are barriers to improving the management and outcomes of chronic pain, including obtaining longitudinal data, assessing response to interventions, and addressing challenges to communication between patients and health care providers (HCPs) [12]. The need to maintain continuity of care for chronic pain patients has also become imperative to avoid treatment disruptions due to public health emergencies, such as COVID-19, limiting in-person visits [13,14].

To bridge this gap, a patient-centric digital health app can be used as a method of remote monitoring to enhance communication between patients and HCPs and promote self-management of patients’ symptoms. While a number of pain apps have been created, they evaluate the biopsychosocial components of pain experiences inadequately and lack clinical involvement [12,15-17]. The scientific validation process for these digital pain apps has not focused on development, adoption, engagement, and patient satisfaction [18-21]. None have been scientifically validated for their impact on pain-related clinical outcomes [16,17]. The few digital pain management solutions that have been scientifically validated for positive clinical impact are specific to patients with lower back pain rather than focused on generalized chronic pain, which is prevalent in a multitude of patients with underlying medical conditions [22]. Furthermore, most studies do not address the effectiveness of mobile pain apps based on clinical setting, despite major lifestyle differences of individuals who live in urban and rural areas [23]. Mobile health apps also have difficulty engaging patients, with top-performing health apps having an average 30-day retention rate of only 15% [24].

A novel digital pain management solution, the Manage My Pain (MMP) app (ManagingLife Inc) [25], was used in this study by patients and HCPs to measure and monitor pain, mental health, and medication use. The purpose of this study was to evaluate the real-world engagement of patients in urban and rural settings in Ontario, Canada with the MMP app alongside their standard of care. Engagement was ascertained by evaluating both adoption and retention rates for continuing use of this app over time. Clinical outcomes of pain and related mental health were also measured and compared between patients who engaged with the app versus those who proceeded with the standard of care at their respective institutions.

## Methods

### Study Sites and Participants

A prospective, open-label, multicenter exploratory study with active and comparator arms was conducted from January 8, 2018, to January 7, 2020, at 3 study sites. Participants were recruited from among new patients with chronic pain conditions who were referred to 2 tertiary academic pain centers in Toronto, Ontario, Canada (Toronto General Hospital [TGH] and Toronto Western Hospital [TWH]) and a rural pain clinic in Ontario (the Iroquois Falls Family Health Team [IFFHT] pain clinic in Iroquois Falls, Ontario). All patients had pain of moderate-to-severe intensity that had persisted for at least three months. All patients, regardless of use of the Manage My Pain app, received the standard of care for the particular clinic, which included interventions such as medication management, psychological therapy, and physiotherapy.

### The Manage My Pain Digital Health Solution

Manage My Pain [26], the app used for this study, is a digital health solution that comprises 3 components: (1) an app for patients to track their pain, function, and medication; respond to questionnaires; and view insights on their conditions; (2) reports that summarize the information collected in the app to be used during clinical visits to facilitate communication between patients and clinicians; and (3) a monitoring portal used by clinics to remotely assess patient progress, assign questionnaires, and highlight clinically relevant trends and patterns using advanced analytics [25]. MMP was first launched in 2011 as the first pain management app on the Android platform. In 2015, ManagingLife partnered with the multidisciplinary team to evolve the solution to meet clinical needs and successfully integrate it into the clinical workflow of an outpatient clinic of an academic hospital [27]. Several papers have applied machine learning techniques to analyze the engagement patterns of users within MMP and develop prediction models from its data set of over 50,000 users [28-30].

**Figure 1:**
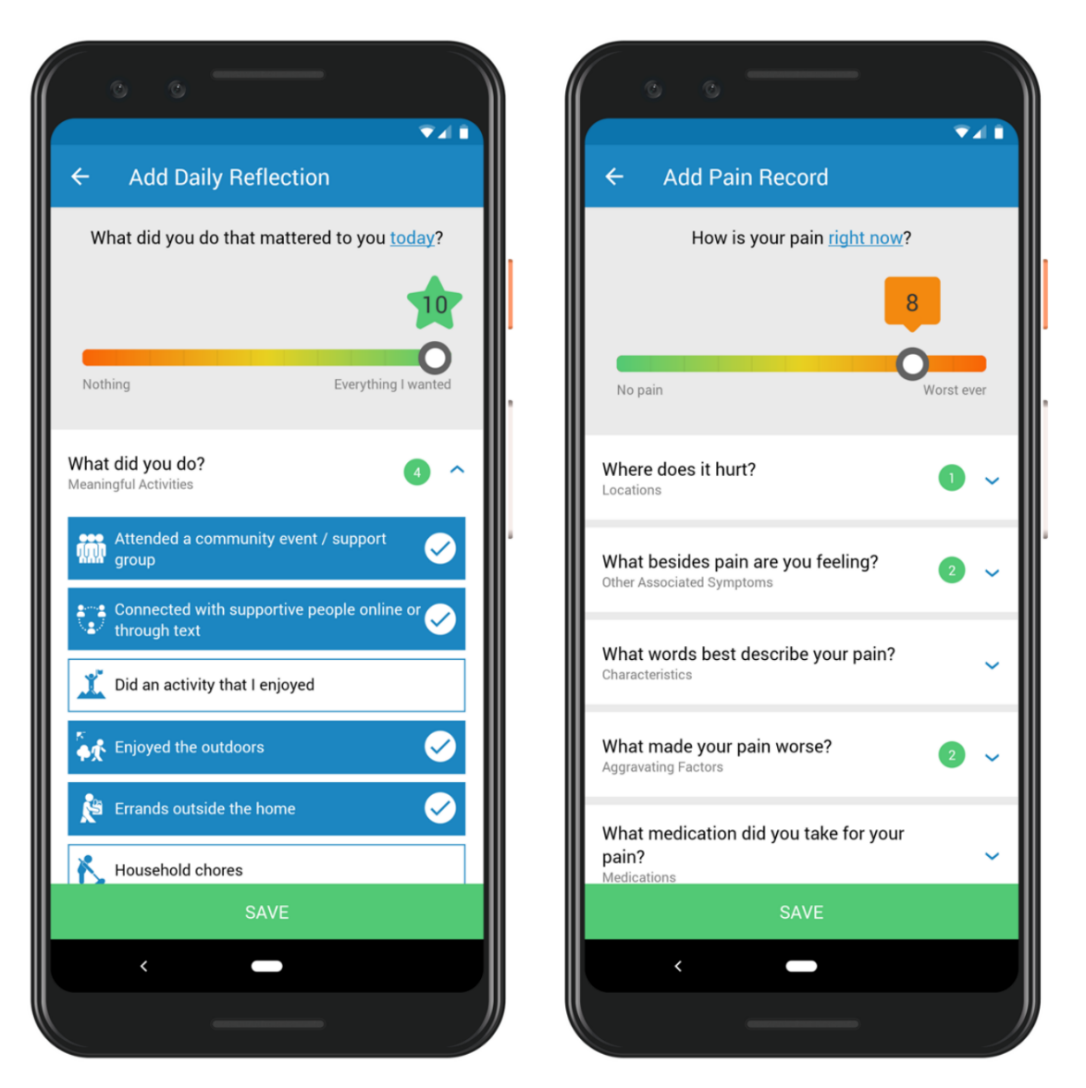
Screenshots of the screens used by Manage My Pain to collect patient-reported outcomes.

MMP is used by both patients and clinics to measure and monitor pain, function, and medication use. Patients can record their experiences by using the MMP app on their mobile device (compatible with Android and iOS devices) or accessing a web-based platform. When prompted by an in-app push notification triggered at 8 PM daily, patients record daily reflections in the app, where they indicate the meaningful activities they were able to accomplish. The daily reflection concept is based on acceptance and commitment therapy principles that have demonstrated an ability to improve clinical outcomes relevant to pain management [27]. Patients also record their pain episodes, including descriptions such as severity, locations, associated symptoms, characteristics, duration, environment, and aggravating or alleviating factors (Figure 1).

**Figure 2:**
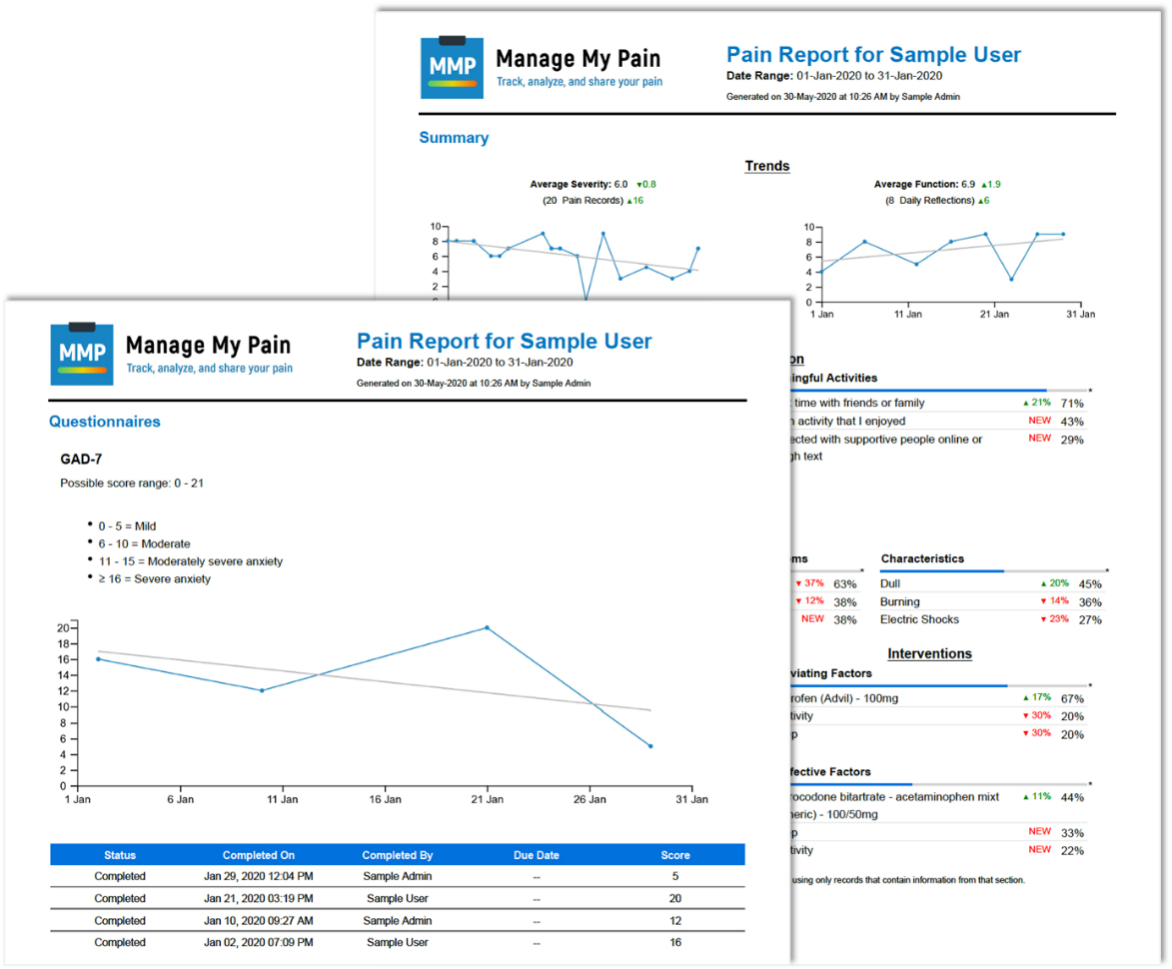
Sample clinician-facing report produced by Manage My Pain that contains scored responses to validated measures as well as a summary of the daily tracking.

Each section of the app can be customized by the patient so that it is representative of their situation. MMP is designed to capture both daily reflections and pain episodes in less than 60 seconds [25]. As patients enter information into the app, charts and graphs are presented to the patient to highlight patterns and trends that increase self-awareness of their condition and provide insight into triggers and interventions. With consent, this information can also be viewed by their clinical team through MMP’s remote monitoring portal. For clinics, pain and function trends are summarized across a predefined time period and can be viewed digitally or output into a clinician-friendly concise report. These self-reported outcomes are used to improve communication with a patient during a clinical visit and assess progress more objectively between clinical visits. Moreover, MMP allows clinics to assign validated questionnaires on pain and related domains for patients to complete at home in advance of their clinical visit. Responses to these questionnaires, along with their corresponding scores and interpretations, are also available through the MMP portal and can be summarized in the clinician-facing report (Figure 2). Emails are sent to the patients by MMP at predefined intervals to encourage engagement with the app and prompt patients to complete the questionnaires by the specified due date.

### Recruitment

As part of the registration process, patients were shown a consent screen asking for permission to share the information from MMP with their clinical team for clinical and research purposes, and they signed an informed consent form. Participants had to explicitly agree by entering a unique randomized 10-digit ID provided to them by the research coordinator, which allowed each clinic to match the participant with their clinical profile and ensure appropriate deidentification. After registration, participants had to activate their account by clicking on a link sent to them by email. Participants were instructed to download MMP through either the Apple App Store (for iPhone users) or the Google Play Store (for Android users) upon successful activation. For participants that did not have either device, a web link associated with their clinic was bookmarked for them to use MMP through their internet browser for easy access.

At first access, the app provided a brief tutorial on how to set up the user’s profile, which included entering relevant medications and pain conditions. It then described how to complete a sample entry by using a touch slider to enter a numerical rating score as an integer from 0 to 10. Once the user’s pain level was indicated, additional questions were prompted, including pain location, other associated symptoms, pain characteristics, aggravating and alleviating factors, and the duration and environment of the pain episode. While all questions included a drop-down menu including prepopulated answers with associated infographics, participants were also given the option to add customized values. A Notes section where participants could enter free text was also provided. Upon completion, patients could save their entry for the patient and health care team to track and observe trends. Technical support was also offered to the participants in the event that they required troubleshooting for using the app, either by the designated research team or the ManagingLife technical support team. Email notifications for data entry on validated questionnaires were sent by the app 7 days prior to the due date set by the research coordinator, which was intended to coincide with the date of the clinical visit. This feature allowed HCPs to track the progress of patients who could not be seen through in-person visits.

### Study Procedures

Participants at the 3 sites were selected using a homogeneous purposive sampling method [31] and given an option to use the app. During the first clinical visit, patients who consented to the study for their data to be collected were offered a choice to either register an account with the app [25] to share their data with the research and clinical teams through the app’s monitoring portal or provide their data on paper-based questionnaires during pain clinic visits. Participants who continued to record their pain experiences and enter data in the app after 30 days of first registering were considered to be in the app group. Participants who declined to use the app or those who registered but had no records in the app after 30 days were considered to be in the nonapp group. A cutoff of 30 days was used based on its acceptance in the mobile app industry as a benchmarked metric of retention [24] and on its determination by the clinical team involved in the study as a meaningful duration of information that could inform clinical decision making.

Participants were asked to complete questionnaires on the following pain-related outcomes: anxiety, depression, pain catastrophizing, pain disability, patient global impression of change, and daily opioid consumption. Daily opioid consumption was measured in oral morphine equivalents in milligrams upon entry into the study during the initial visit and as a part of the first follow-up clinical visit within 4.5 months of the initial visit (short-term follow-up) and during the second follow-up clinical visit between 4.5 and 7 months after the initial visit (long-term follow-up). Given that our objective was to validate the impact of the app in a real-world clinical environment, the date ranges of follow-up visits were more broadly defined to align with the date of the actual clinical visit. Clinicians at each participating site were encouraged to use the clinical reports, either digitally through the portal or printed, during their clinical visits with the patients.

Participants who agreed to use the app but did not have in-person clinical appointments within these time frames were still remotely prompted to complete the questionnaires through the app portal by research staff. Patients in the nonapp group provided their data only by completing paper-based questionnaires during clinic visits or via a phone interview if no in-person clinical visit was scheduled during study-related follow-ups.

### Validated Measures Used in the Study

The feasibility and successful adoption of the digital health solution was evaluated through clinical outcomes and patient engagement. During the initial visit, participants completed baseline questionnaires that were standardized across sites as well as questionnaires that were considered the standard of care at each individual clinical practice.

As mood disorders are often prevalent in individuals with chronic pain and have been known to affect and intensify pain perception [32,33], anxiety and depression levels were recorded using the Generalized Anxiety Disorder 7-item questionnaire (GAD-7) and the Patient Health Questionnaire 9-item scale (PHQ-9) for depression, respectively. These questionnaires have repeatedly demonstrated excellent test-retest reliability [34], criterion and construct validity [35], and high levels of specificity and sensitivity in the assessment of anxiety and depression in patients with chronic pain [34-38]. In addition, the score on the Pain Disability Index (PDI), a 7-item instrument used to evaluate the degree of pain-related disability, can be inversely correlated with overall function [39]. The Pain Catastrophizing Scale (PCS) was also administered to participants to measure the degree of maladaptive cognitive distortions known as catastrophizing, which increase negative emotional schema throughout the anticipation and experience of painful stimulation [40,41]. Additionally, the Patient Global Impression of Change (PGIC) questionnaire, a 7-level ordinal measure, was administered to participants at both short-term and long-term follow-up time frames to assess the degree of improvement or worsening of a patient’s clinical condition. The PGIC is a single-item validated questionnaire that asks the user about their perceived improvement and is significantly correlated with changes in pain intensity, efficacy of treatment, and interference of pain in daily activities [42].

Finally, participants’ opioid consumption was measured over time using oral morphine equivalents (OME) through conversion ratios outlined by the Canadian Guideline for Safe and Effective Use of Opioids for Chronic Non-Cancer Pain [43]. Given that the core functionality of Manage My Pain is to record pain intensity scores during each engagement with the app, it was not selected as a measure for evaluation, as any comparisons against patients recording this information using point-in-time questionnaires would be misleading. Specifically, other measures were selected to assess the mental and physical well-being of patients independent of their severity and intensity.

### Ethics Approval

Institutional ethics board approvals were obtained from each study site by the University Health Network Research Ethics Board for the academic sites (TGH and TWH) and Veritas Institutional Review Board for the rural site (IFFHT). The approval process involved confirmation that MMP has the administrative and technical safeguards in place to ensure compliance with privacy legislation.

### Statistical Analyses

Continuous data were summarized using mean and standard deviation or median and interquartile ranges, and categorical data were summarized using frequency and percentages. Univariate tests for continuous data were conducted using 2-sample *t* tests or Wilcoxon rank sum tests as appropriate based on the distribution of the data. Chi-square tests or Fisher exact tests were used for categorical data. A random-effects model was used for all outcomes to account for correlations arising from repeated measures within the same individual.

The main exposure of interest was whether someone used the app for at least 30 days, adjusted for time, age, gender, and study site. Each of the 6 outcomes (daily OME, GAD-7 score, PHQ-9 score, PDI score, PCS score, and PGIC score) was also modeled to examine the association of the intervention (use of the app for at least 30 days) with the outcome after controlling for other relevant variables. The duration of usage (time) among the participants who entered data into the app was recorded as the difference between the most recent entry and the date of registration. An interaction term between time (short term or long term) and group (intervention or control) was examined to determine whether the intervention was associated with differences between groups over time. A likelihood ratio test was used to assess the statistical significance of the interaction term, and the term was included in the model if it remained statistically significant at the .05 significance level. For clinical utility, the primary analysis categorized time as baseline, short term, and long term. A sensitivity analysis was conducted using time as a continuous covariate of interest. The baseline value was adjusted for by including it in the outcome vector [44]. Model checking for continuous outcomes was performed using analysis of residuals. Bootstrapped 95% confidence intervals and *P* values were provided where the model residuals violated the normality assumption.

## Results

### Data at Baseline and Engagement With the App

**Figure 3:**
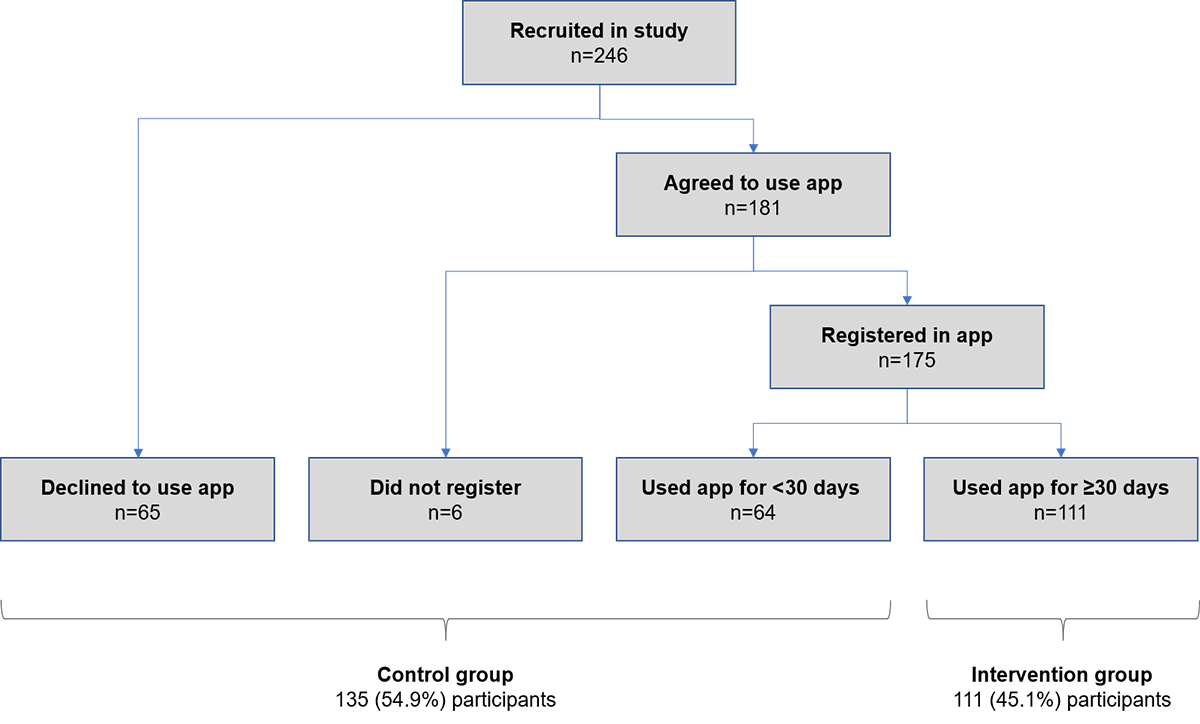
Flow diagram of group allocation based on participants’ engagement with the app.

The average age of participants in the study was 56.67 (SD 13.12) years, with 60.2% (148/246) of participants being female. A total of 246 participants were enrolled across the 3 sites (154 participants at the 2 urban sites and 92 participants at the rural site), out of which 181 (73.6%) accepted the use of the app in their clinical care and the remaining 65 (26.4%) continued with paper-based data entry at their respective clinics. Of the 181 participants who agreed to use the app, 175 (96.7%) participants registered and provided consent to share their data. Of the 175 participants who registered, 111 (63.4%) participants used the app for at least 30 days and therefore were considered part of the intervention (app) group (Figure 3). Data from 70 participants who initially accepted the use of the app but either did not use the app or used it for less than 30 days were combined with data from the 65 participants who had declined to use the app at the start of the study, and these 135 participants were considered to be in the nonapp group for analysis (Figure 3). There were no differences between the app and nonapp groups with respect to demographics, duration of pain, or the validated measures for mood and physical disability (Table 1).

**Table 1:** Baseline demographics characteristics of the study population.

Characteristic		Nonapp group^a^ (n=135)	App group (n=111)	*P* value
Age (years), mean (SD)		57.17 (15.07)	56.05 (10.31)	.51
Male sex, n (%)		59 (44.0)	39 (35.1)	.20
**Employment status, n (%)**				.69
	Never worked	1 (0.8)	0 (0.0)	
	Not working	58 (45.3)	51 (46.4)	
	Working (part- or full-time)	27 (21.1)	27 (24.5)	
	Retired	42 (32.8)	32 (29.1)	
**Duration of pain, n (%)**				.16
	<12 months	7 (5.3)	13 (11.9)	
	12-24 months	13 (9.8)	12 (11.0)	
	>24 months	112 (84.8)	84 (77.1)	
**Etiology of pain, n (%)**				.005
	Accident	35 (26.9)	35 (31.8)	
	As a result of illness	27 (20.8)	11 (10.0)	
	Following surgery	13 (10.0)	8 (7.3)	
	No known reason	43 (33.1)	54 (49.1)	
	Other	12 (9.2)	2 (1.8)	
GAD-7^b^ score, mean (SD)		8.51 (6.21)	8.21 (6.35)	.71
PHQ-9^c^ score, mean (SD)		11.26 (6.83)	11.52 (6.66)	.77
PDI^d^ score, mean (SD)		40.30 (16.47)	41.76 (15.11)	.49
PCS^e^ score, mean (SD)		23.84 (13.19)	24.93 (14.39)	.55
OME^f^ (mg/24 hr), median (IQR)		0 (0-30)	0 (0-27)	.42

^a^The nonapp group included data from the 65 participants who had declined to use the app at the start of the study and the 70 participants who initially accepted use of the app but either did not use the app or used it for less than 30 days.

^b^GAD-7: Generalized Anxiety Disorder 7-item scale.

^c^PHQ-9: Patient Health Questionnaire 9-item scale.

^d^PDI: Pain Disability Index.

^e^PCS: Pain Catastrophizing Scale.

^f^OME: oral morphine equivalents.

Of those who used the app for at least 30 days, the mean number of records entered was 113.4 (SD 129.7). The mean duration of usage from the date of registration to the date of the last record entered was 164.2 (SD 88.4) days.

### Clinical Outcomes

Quantitative analysis of the clinical outcomes was performed at 2 time points, short term and long term, following enrollment into the study. Of the 135 patients in the nonapp group, 36 (26.7%) provided their data at the short-term follow-up and 31 (23.0%) provided their data at the long-term follow-up. A total of 90 of the 111 participants in the app group provided their data through the app for the short-term follow-up, and 69 provided their data for both short- and long-term follow-ups (Table 2). The primary reason for the large number of patients whose data were not available at the short-term follow-up is that many were deemed not to qualify for treatments offered at the clinics shortly after consenting to participate in the study and therefore were discharged from the clinic. The patients were discharged because these clinics accepted only patients with pain whose condition was amenable to the interventions offered at the clinic (eg, high-dose intravenous ketamine infusions, neuromodulation implants). For each of the measures collected from participants at the short-term and long-term follow-ups, less than 5% of data were missing for each time point, and no imputation technique was used.

The number of elapsed days from baseline for both the short-term and long-term follow-ups was not significantly different across the intervention and control groups (Table 3).

**Figure 4:**
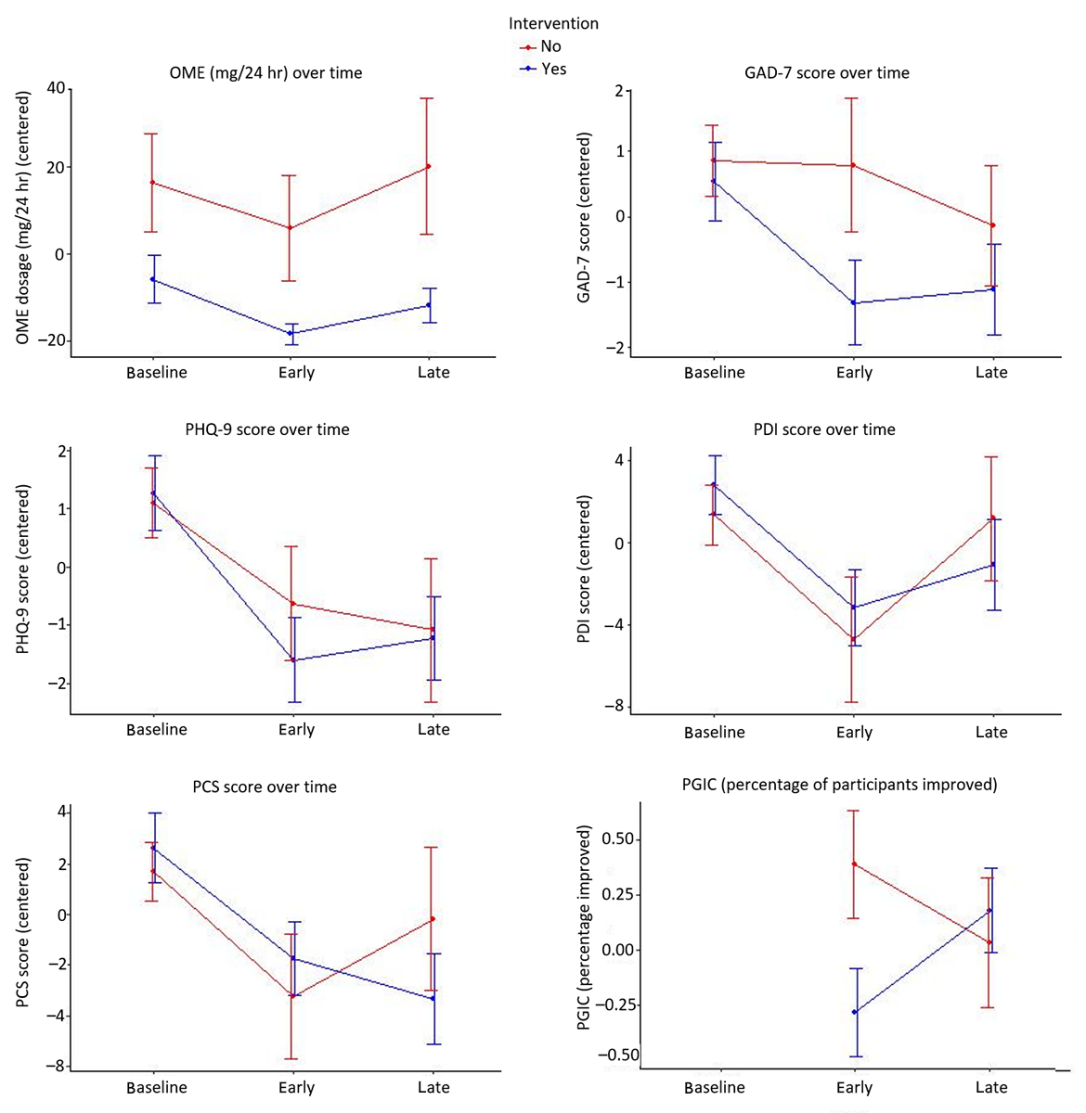
Mean values of validated measures in both app (labelled "Intervention – Yes") and nonapp (labelled "Intervention – No") groups at short-term (early) and long-term (late) follow-ups. Error bars indicate the standard error. Means have been centered along the overall mean. GAD-7: Generalized Anxiety Disorder 7-item scale; OME: oral morphine equivalence; PCS: Pain Catastrophizing Scale; PDI: Pain Disability Index; PGIC: Participant Global Impression of Change; PHQ-9: Patient Health Questionnaire 9-item scale.

Unadjusted analyses did not find any significant differences between the intervention and control groups over time (Table 4 and Figure 4).

**Table 2:** Number of participants with questionnaire responses at the short-term follow-up (prior to 4.5 months from baseline) and the long-term follow-up (between 4.5 and 7 months from baseline).

Group		Participants, n	Baseline, n (%)	Short-term, n (%)	Long-term, n (%)
**Nonapp**		135	130 (96.3)	36 (27.7)	31 (23.8)
	Declined to use app	65	64 (98.5)	22 (34.4)	19 (29.7)
	Did not register	6	3 (50.0)	2 (66.7)	2 (66.7)
	Used app for <30 days	64	63 (98.4)	12 (19.0)	10 (15.9)
App (used app for ≥30 days)		111	111 (100.0)	90 (81.1)	69 (62.2)
Total		246	241 (98.0)	126 (52.3)	100 (41.5)

**Table 3:** Days from baseline for both short-term and long-term follow-up time periods. *P* values used a Wilcoxon test and 95% CIs were bootstrapped.

Time	App group, median (IQR) (n=111)	Nonapp group, median (IQR) (n=135)	Difference (95% CI)	*P* value
Short-term	92 (80-100)	91 (78-104)	1 (–7 to 8)	.80
Long-term	183 (162-197)	188.5 (168-194)	–5.5 (–18 to 2)	.83

**Table 4:** Unadjusted outcomes stratified by time and group. The differences between the intervention and control groups are represented as absolute numbers with their 95% CIs, and 95% CI for OME used bootstrapped values.

Time		App group	Nonapp group	Difference (95% CI)	*P* value
**OME^a^ (mg/24 hr), median (IQR)**					
	Baseline	0 (0-27)	0 (0-30)	0 (–14 to 0)	.39
	Short-term	0 (0-15)	0 (0-27)	0 (–30 to 0)	.18
	Long-term	0 (0-23)	2 (0-73)	–2 (–71 to 0)	.16
**GAD-7^b^ score, mean (SD)**					
	Baseline	8.21 (6.35)	8.53 (6.23)	–0.32 (–1.95 to 1.30)	.70
	Short-term	6.34 (6.17)	8.46 (6.13)	–2.12 (–4.58 to 0.35)	.09
	Long-term	6.54 (5.86)	7.53 (5.14)	–0.99 (–3.34 to 1.36)	.43
**PHQ-9^c^ score, mean (SD)**					
	Baseline	11.52 (6.66)	11.35 (6.78)	0.17 (–1.57 to 1.91)	.85
	Short-term	8.66 (6.93)	9.63 (5.92)	–0.97 (–3.46 to 1.53)	.47
	Long-term	9.03 (5.88)	9.17 (6.84)	–0.14 (–3.02 to 2.75)	.92
**PDI^d^ score, mean (SD)**					
	Baseline	41.76 (15.11)	40.32 (16.53)	1.44 (–2.67 to 5.55)	.49
	Short-term	35.78 (17.57)	34.23 (18.37)	1.55 (–5.74 to 8.83)	.67
	Long-term	37.90 (18.32)	40.14 (16.74)	–2.24 (–10.01 to 5.52)	.58
**PCS^e^ score, mean (SD)**					
	Baseline	24.93 (14.39)	24.00 (13.13)	0.93 (–2.65 to 4.51)	.61
	Short-term	20.58 (13.77)	19.06 (14.84)	1.52 (–4.32 to 7.36)	.59
	Long-term	18.97 (14.94)	22.13 (15.80)	–3.16 (–10.00 to 3.67)	.35
**PGIC^f^ score^g^, n/N (%)**					
	Baseline	N/A^h^	N/A	N/A	N/A
	Short-term (improved)	32/89 (36.0)	15/32 (46.9)	–10.9% (–33.0% to 11.1%)	.38
	Long-term (improved)	24/58 (41.4)	11/28 (39.3)	2.1% (–22.1% to 26.3%)	>.99

^a^OME: oral morphine equivalents.

^b^GAD-7: Generalized Anxiety Disorder 7-item scale.

^c^PHQ-9: Patient Health Questionnaire 9-item scale.

^d^PDI: Pain Disability Index.

^e^PCS: Pain Catastrophizing Scale.

^f^PGIC: Patient Global Impression of Change.

^g^PGIC score represents participants who improved.

^h^N/A: not applicable.

### Adjusted Analysis for the Entire Study Cohort

A significant decline in daily OME in milligrams was observed in both the short-term (decrease of 8.31 mg, 95% CI –16.62 to –0.97) and long-term (decrease of 12.59 points, 95% CI –21.16 to 4.27) time periods when compared with baseline. Depression (PHQ-9) (lower by 2.29 points, 95% CI –3.23 to –1.34 in the short-term follow-up; lower by 2.52 points, 95% CI –3.56 to –1.47 in the long-term follow-up) and disability (PDI) scores (lower by 5.20 points, 95% CI –7.60 to –2.81 in the short-term follow-up; lower by 3.52 points, 95% CI –6.20 to –0.80 in the long-term follow-up) decreased significantly for all participants (Multimedia Appendix 1). Pain Catastrophizing Scale scores decreased at the short-term follow-up, with the scores lowered by 3.53 points (95% CI –6.88 to –0.17) but returned to baseline at the long-ter m follow-up. Older participants reported lower opioid use over time, with a decrease of 0.98 mg of OME per year of increasing age (95% CI –1.80 to –0.08). Increasing age was also associated with lower GAD-7, PHQ-9, PDI, and PCS scores (Multimedia Appendix 1). Male sex was associated with a higher disability score throughout the study (Multimedia Appendix 1).

Participants in the app group had lower anxiety (GAD-7) scores at the short-term follow-up (decrease of 2.10 points, 95% CI –3.96 to –0.24) and lower Pain Catastrophizing Scale scores at the long-term follow-up (decrease of 5.23 points, 95% CI –9.55 to –0.91) (Multimedia Appendix 1). For the reduction in the anxiety and pain catastrophizing scores, there was a significant intervention-by-time interaction, indicating that the decrease in these scores was higher in the group that used the app. There was also a change over time for the daily OME (lower by 12.59 mg, 95% CI –21.16 to –4.27), PHQ-9 score (decrease of 2.52 points, 95% CI –3.56 to –1.47), and PDI score (decrease of 3.52 points, 95% CI –6.20 to –0.80), but there was no time-by-intervention interaction, indicating that the change in outcome over time was not different between those who used the app and those who did not.

## Discussion

### Summary of the Main Results of the Study

This is the first multisite study at rural and urban pain clinics of a digital pain management solution that compared outcomes in patients with chronic pain who chose to use the app in addition to standard care versus those who received only standard care. A total of 73.6% (181/246) of the participants in the study chose to enroll for the app, 45.1% (111/246) continued to use it beyond one month following enrollment, and 28.0% (69/246) were still using it for 4.5 to 7 months. There was evidence of a decrease in anxiety and pain catastrophizing in participants who used the app versus those who did not use the app.

### Acceptance of and Engagement With the App

Digital health applications can play a significant role in enhancing the connectivity between patients and their HCPs. Though some studies report men and younger age groups as more likely to engage with this kind of technology [45], our study and others did not find differences based on age and sex [45,46]. It is possible that the chronicity of pain in participants in our study and the lack of effective therapies made patients in our study interested in exploring the potential for help from the app offered in our study. The rates of initial engagement with the app reported in our study—initially 73.6% (181/246), with a gradual decrease to 28.0% (69/246) at the long-term follow-up—are consistent with those reported in literature by our group [47] and others [45,48,49] and appear to be better than the rates for other apps [24]. We measured ongoing engagement with the digital app, unlike other studies that evaluate merely the intent of patients to engage with digital health solutions [46]. Continuing engagement with digital health solutions is important, and international health organizations have also emphasized the importance of developing evidence for the integration of digital health solutions in routine medical care [27,28] because of their potential to empower and enable patients. A follow-up study will focus on the engagement patterns and their contributing factors within this study along with their potential correlations with the clinical outcomes seen.

### Association of Using the App With Pain-Related Clinical Outcomes

Our study found that use of the app was associated with a reduction in pain-related anxiety and pain catastrophizing scores. These reductions have clinical significance, given that the minimum clinically important difference in values for the GAD-7 and PCS (–4 for GAD-7 [50] and 38% for PCS [51]) is within the 95% confidence interval of the outcome reduction. Pain-related anxiety and catastrophizing can have significant adverse effects on patients, with an increase in both health care use [52] and the probability of misuse of prescription opioids [53].

The ability of patients to track and reflect on their pain and its relationship to activities and medications in our study may have resulted in an attenuation of the psychological correlates of chronic pain. Self-monitoring of symptoms is an important component of most pain self-management programs [54]. There is a growing body of evidence that self-monitoring using eHealth tools is associated with positive health outcomes [55]. In particular, the daily reflection concept used by Manage My Pain is a form of self-monitoring based on acceptance and commitment therapy principles, which is an empirically supported treatment for individuals living with chronic pain [56]. It emphasizes engagement in meaningful activities based on personal values as a cornerstone of treatment [57]. The use of the app’s diary of patients’ lived experiences when interacting with HCPs through reports or the remote monitoring portal possibly empowered patients to address their negative emotions. This empowerment has been known to be associated with an analgesic benefit over time in patients with chronic pain [58,59]. Studies on other chronic health conditions have also reported similar results [60]. While the differences in the other clinical domains between the intervention and control groups were not significant, it is possible that these differences would be significant if the sample size were larger. Further, we did not include pain intensity scores in our study because this instrument has been shown to lack the ability to demonstrate functional benefits of analgesic interventions [61].

This study suggests that engagement by patients with an app-based digital pain solution that incorporates validated questionnaires may be associated with improvement in clinical outcomes. A future study will present the results of a qualitative analysis that assessed both the patients’ and clinical team’s perspective on the app’s utility. Additionally, further research is required to understand factors that impact initial acceptance and continuing engagement with digital apps in patients with pain, including user comfort, understanding of technology, accessibility for patients and connectivity with HCPs, and feasibility of implementation in established health care systems [62].

### Limitations of This Study

This study of the outcomes of the use of an app-based digital solution for generalized chronic pain has some limitations. Participants in this study were not allocated to study groups by randomization. This could have introduced a bias because patients comfortable with digital technology were more likely to opt to use the app. An additional bias could have been introduced in that patients who chose to use the app would have seen clinical improvements regardless of app usage. The high drop-off rate from baseline to the short-term follow-up with the control group may have also introduced a bias in the results. However, the fact that that a large number of patients in this control group were deemed not to qualify for treatments offered at the clinics and were therefore discharged implies that they would not have benefited relative to those at least receiving the standard of care. This may have also contributed to a higher dropout rate for using the app. All measures were based on participant self-reports, which were not verified by objective means (eg, clinical interview to assess anxiety and depressive disorders, verification of opioid use by pharmacy records). This may have resulted in biased estimates of results that differed by treatment group, confounding the present findings.

### Conclusions

This study of a novel digital pain management solution that incorporated validated measures for domains of pain in patients at urban and rural clinics found that 28.0% (69/246) of all patients continued to use the app on a long-term basis. Patients that engaged with the digital health solution had less anxiety and lower pain catastrophizing scores as measured by validated tools. Digital pain management applications and other health-related clinical applications deserve significant attention in the years ahead, given the push toward mobile health tools and telemedicine.

## Appendix

Multimedia Appendix 1 Results of the adjusted analysis.

## Abbreviations

GAD-7: Generalized Anxiety Disorder 7-item scale
HCP: health care provider
IFFHT: Iroquois Falls Family Health Team
MMP: Manage My Pain
OME: oral morphine equivalents
PCS: Pain Catastrophizing Scale
PDI: Pain Disability Index
PGIC: Patient Global Impression of Change
PHQ-9: Patient Health Questionnaire 9-item scale
TGH: Toronto General Hospital
TWH: Toronto Western Hospital
