# Supplementary material for: User Engagement and Clinical Impact of the Manage My Pain App in Patients With Chronic Pain: A Real-World, Multi-site Trial
Source: JMIR Mhealth Uhealth. 2021 Mar 4;9(3):e26528. doi: 10.2196/26528 (PMC7974758; doi:10.2196/26528)
Supplement: Multimedia Appendix 1 [file mhealth_v9i3e26528_app1.docx]

Table 5. Results of the adjusted analysis. Significant values are italicized and shaded. OR = odds ratio, NA = not applicable; NS = not significant; CI= confidence interval. Each column displays the results of each outcome. The PGIC results are displayed using odds ratios (Improvement versus reference = worse/ no change).

|  | **OME - mg/24h (95% CI)** | **GAD7 Score (95% CI)** | **PHQ9 Score (95% CI)** | **PDI Score (95% CI)** | **PCS Score (95% CI)** | **PGIC Short-Term Score* (95% CI)** | **PGIC Long-Term Score*  (95% CI)** |
| --- | --- | --- | --- | --- | --- | --- | --- |
| **Age** | *-0.98 (-1.80 to -0.08)* | *-0.14 (-0.19 to -0.08)* | *-0.14 (-0.20 to -0.08)* | *-0.19 (-0.35 to -0.04)* | *-0.17 (-0.30 to -0.04)* | 1.03 (0.99, 1.08) | 0.99 (0.94, 1.04) |
| **Gender Male vs. Female** | -1.98 (-26.06 to 22.60) | 0.10 (-1.38 to 1.59) | -0.21 (-1.81 to 1.38) | *4.69 (0.53 to 8.84)* | 2.58 (-0.91 to 6.08) | 1.75 (0.56, 5.49) | 0.83 (0.28, 5.43) |
| **Time Period (Baseline)** | | | | Ref | | | |
| **Time Period (Short-Term)** | *-8.31 (-16.62 to -0.97)* | 0.59 (-0.97 to 2.14) | *-2.29 (-3.23 to -1.34)* | *-5.20 (-7.60 to -2.81)* | *-3.53 (-6.88 to -0.17)* | NA | NA |
| **Time Period (Long-Term)** | *-12.59 (-21.16 to -4.27)* | -0.38 (-2.02 to 1.27) | *-2.52 (-3.56 to -1.47)* | *-3.52 (-6.20 to -0.80)* | -0.34 (-3.88 to 3.20) | NA | NA |
| **App group vs. Non-app group** | -12.64 (-37.32 to 11.46) | -1.15 (-2.86 to 0.56) | -0.57 (-2.33 to 1.18) | 2.05 (-2.49 to 6.60) | -0.06 (-4.02 to 3.90) | 1.06 (0.25, 4.42) | 0.56 (0.16, 1.98) |
| **Intervention x Short-Term** | NS | *-2.10 (-3.96 to -0.24)* | NS | NS | 0.63 (-3.40 to 4.63) | NA | NA |
| **Intervention x Long-Term** | NS | -1.00 (-3.01 to 1.00) | NS | NS | *-5.23 (-9.55 to -0.91)* | NA | NA |

GAD-7: Generalized Anxiety Disorder 7-item Scale; OME: Oral Morphine Equivalence; PCS: Pain Catastrophizing Scale; PDI: Pain Disability Index; PHQ-9: Patient Health Questionnaire 9-item Scale
* The PGIC score was dichotomized as impression of any improvement in chronic pain versus no change or worsening and the nature of the question required the short-term and long-term time periods to be analyzed separately
